# Enhanced left superior parietal activation during successful speech production in patients with left dorsal striatal damage and error-prone neurotypical participants

**DOI:** 10.1093/cercor/bhac282

**Published:** 2022-08-13

**Authors:** Sharon Geva, Letitia M Schneider, Shamima Khan, Diego L Lorca-Puls, Andrea Gajardo-Vidal, PLORAS team, Storm Anderson, Rachel Bruce, Megan Docksey, Kate Ledingham, Louise Lim, Sophie Roberts, Thomas M H Hope, David W Green, Cathy J Price

**Affiliations:** Wellcome Centre for Human Neuroimaging, Institute of Neurology, University College London, 12 Queen Square, London WC1N 3AR, United Kingdom; Wellcome Centre for Human Neuroimaging, Institute of Neurology, University College London, 12 Queen Square, London WC1N 3AR, United Kingdom; Department of Cognition, Emotion, and Methods in Psychology, Faculty of Psychology, University of Vienna, Universitätsring 1, 1010 Vienna, Austria; Wellcome Centre for Human Neuroimaging, Institute of Neurology, University College London, 12 Queen Square, London WC1N 3AR, United Kingdom; Wellcome Centre for Human Neuroimaging, Institute of Neurology, University College London, 12 Queen Square, London WC1N 3AR, United Kingdom; Sección Neurología, Departamento de Especialidades, Facultad de Medicina, Universidad de Concepción, Victor Lamas 1290, Concepción, 4030000, Chile; Wellcome Centre for Human Neuroimaging, Institute of Neurology, University College London, 12 Queen Square, London WC1N 3AR, United Kingdom; Faculty of Health Sciences, Universidad del Desarrollo, Ainavillo 456, Concepción, 4070001, Chile; Wellcome Centre for Human Neuroimaging, Institute of Neurology, University College London, 12 Queen Square, London WC1N 3AR, United Kingdom; Wellcome Centre for Human Neuroimaging, Institute of Neurology, University College London, 12 Queen Square, London WC1N 3AR, United Kingdom; Department of Experimental Psychology, Faculty of Brain Sciences, University College London, 26 Bedford Way, London, WC1H 0AP, United Kingdom; Wellcome Centre for Human Neuroimaging, Institute of Neurology, University College London, 12 Queen Square, London WC1N 3AR, United Kingdom

**Keywords:** aphasia, fMRI, putamen, recovery, stroke

## Abstract

Functional imaging studies of neurotypical adults report activation in the left putamen during speech production. The current study asked how stroke survivors with left putamen damage are able to produce correct spoken responses during a range of speech production tasks. Using functional magnetic resonance imaging, activation during correct speech production responses was assessed in 5 stroke patients with circumscribed left dorsal striatal lesions, 66 stroke patient controls who did not have focal left dorsal striatal lesions, and 54 neurotypical adults. As a group, patients with left dorsal striatal damage (our patients of interest) showed higher activation than neurotypical controls in the left superior parietal cortex during successful speech production. This effect was not specific to patients with left dorsal striatal lesions as we observed enhanced activation in the same region in some patient controls and also in more error-prone neurotypical participants. Our results strongly suggest that enhanced left superior parietal activation supports speech production in diverse challenging circumstances, including those caused by stroke damage. They add to a growing body of literature indicating how upregulation within undamaged parts of the neural systems already recruited by neurotypical adults contributes to recovery after stroke.

## Introduction

The putamen is involved in several interacting cortico-striatal circuits, each subserving different functions (Haber 2016). It receives rich input from all cerebral lobes and sends output back to the cortex via the globus pallidus and the thalamus (Alexander et al. 1986). The left putamen and the caudate nucleus (which together form the dorsal striatum) contribute to a wide range of motor and cognitive functions including speech and language processing (Murdoch 2001). Although functional imaging studies of healthy adults have provided evidence for involvement of the putamen in language and speech production (Viñas-Guasch and Wu 2017), group studies of stroke survivors have not consistently associated left dorsal striatal damage with the occurrence and/or type of aphasia (D’esposito and Alexander 1995; Russmann et al. 2003; Komiya et al. 2013). Likewise, lesion-symptom mapping studies have produced mixed results (Colombo et al. 1989; Kang et al. 2017; Kim et al. 2021). These inter-study discrepancies may be attributable to a number of factors, such as inter-patient variability in symptoms within each aphasic syndrome (Bates et al. 2005) and/or the speed and degree of recovery, which in turn may be the consequence of other variables such as the degree of damage to surrounding areas and/or premorbid factors.

When language impairments are observed in stroke survivors with focal damage to the left dorsal striatum, they generally take the form of impaired speech production with preserved comprehension (Alexander and Loverme 1980; Brunner et al. 1982; Damasio et al. 1982; Wallesch et al. 1983; Mega and Alexander 1994; Troyer et al. 2004). The symptoms reported include word finding difficulty (Alexander and Loverme 1980; Brunner et al. 1982; Wallesch et al. 1983; Troyer et al. 2004), paraphasias (Brunner et al. 1982; Damasio et al. 1982; Wallesch et al. 1983), and reduced fluency (Mega and Alexander 1994; Troyer et al. 2004), with dysarthria observed in some studies (Alexander and Loverme 1980; Damasio et al. 1982; Mega and Alexander 1994), but not others (Brunner et al. 1982; Wallesch et al. 1983). Supporting these results from studies of stroke survivors, intraoperative direct electrical stimulation in patients with gliomas demonstrated that stimulation of the dominant anterior putamen causes speech arrest, whereas stimulation of the head of caudate nucleus causes perseverations during picture naming (Robles et al. 2005).

The effect of dorsal striatal damage on speech production observed in some patients raises the following question: how do stroke survivors with left putamen damage successfully produce speech? One possibility is that undamaged regions compensate for the lost function by upregulating their activation. Such upregulation can occur either (i) within the “normal” network, in which case upregulated regions would also be activated in neurotypical participants, or, (ii) in new regions, not routinely used for the specific function pre-stroke.

In principle, functional imaging allows us to address the question of how some stroke survivors with left putamen damage are able to produce correct responses during speech production tasks. Several single and multiple case studies have used functional magnetic resonance imaging (fMRI) to investigate word production in stroke patients with left subcortical damage (Kim et al. 2002; Fridriksson et al. 2005; Abutalebi et al. 2009; Seghier et al. 2014). However, evidence of abnormal modulation of the system that supports language processing requires the inclusion of neurotypical adult controls, and only 2 case studies meet this criterion (Fridriksson et al. 2005; Seghier et al. 2014). The first (Fridriksson et al. 2005) presented a case study of a patient with foreign accent syndrome following focal left putamen damage and revealed increased activation, compared with neurotypical controls (NC), in the left central sulcus and left ventral angular gyrus during overt picture naming. The second (Seghier et al. 2014) presented a case study of a patient with damage to the left dorsal striatum and adjacent white matter and showed higher than normal activation in, and connectivity with, the left premotor cortex when overtly reading words and naming pictures (Seghier et al. 2014). The small number of studies and participants, and the inconsistency in the results across studies, make it difficult to generate specific anatomical predictions for the current study. And while both studies show evidence of upregulation within the network activated during language processing in neurotypical adults, they did not report how consistently this upregulation is observed in the presence versus absence of stroke damage to the putamen.

Using fMRI, we examined the brain regions that support correct responses during a variety of overt speech production tasks in 5 stroke survivors with damage to the left dorsal striatum (i.e. including the putamen) compared with 54 NC and 66 stroke survivors who did not have focal left striatal damage. First, we identified which parts of the brain show abnormally high or low activation in patients with focal left dorsal striatal damage compared with NC, during 11 different speech production tasks and 2 semantic decision tasks. Regions showing “abnormal activation” during correct (accurate) speech production responses became our regions of interest (ROI). The various tasks allowed us to identify activation associated with different levels of articulatory demands in Experiment 1, or stimulus modality, verbal input, and semantic input in Experiment 2. This was motivated by the fact that such linguistic parameters are known to affect speech production (Forster and Chambers 1973; Vitevitch 2002; Saletta et al. 2016) and have been previously associated with language-related dorsal striatal function (e.g. Tettamanti et al. 2005; Thames et al. 2012).

Second, we assessed how specific the abnormal effects were to patients with left focal dorsal striatal damage by comparing ROI activation, during accurate speech production, in patients with a wide range of different lesion sites. Third, we examined the normal function of the ROIs by comparing ROI activation during 13 different tasks in our NC group. Finally, we examined whether ROI activation, during accurate speech production, was sensitive to how easily NC could perform the in-scanner speech production tasks. This involved splitting the NC into 2 groups, based on accuracy levels. If ROI activation is higher in NC who make more versus less errors, then it may be related to increased demands on an error-prone speech production system. Such a result would be in keeping with other studies that have highlighted the role of executive mechanisms in language recovery (Geranmayeh et al. 2017), and findings showing that recovery is mediated by the same network that supports task performance in neurotypical adults (Stefaniak et al. 2021, 2022).

## Materials and methods

### Participants

Patients were recruited from the Predicting Language Outcome and Recovery After Stroke (PLORAS) database, which records behavioral, demographic, and imaging data from participants with a history of adult stroke (Seghier et al. 2016). Cognitive and language abilities were assessed using the Comprehensive Aphasia Test (CAT; Swinburn et al. 2004), which includes a 6-task cognitive screen and 21 speech and language tests. If their speech production abilities were sufficiently good, and they were able to tolerate the magnetic resonance imaging (MRI) environment, patients were invited to complete an fMRI study at a later date.

**Table 1:** Demographics and testing details for the POI.

**Patient ID**	**Lesion location**	**Age at stroke**	**Sex**	**Lesion volume**	**Stroke to CAT**	**Stroke to fMRI**	**CAT to fMRI**
PS0619	L putamen, internal capsule superior longitudinal fasciculus	54	M	45.94	4.5	9.9	5.4
PS0883	L putamen and part of head of caudate nucleus	48	F	9.93	1.7	1.9	0.2
PS2057	L putamen	25	M	11.10	21.2	22.9	1.7
PS2371	L anterior putamen	58	M	6.98	2.8	4.6	1.8
PS2439	L lateral putamen and body of caudate nucleus	68	F	3.76	2.9	4.6	1.7

Patient ID: from the PLORAS database, Sex = male (M) or female (F), lesion volume in cm^3^, “stroke to fMRI,” “stroke to CAT,” and “CAT to fMRI” are measured in years.

**Fig. 1:**
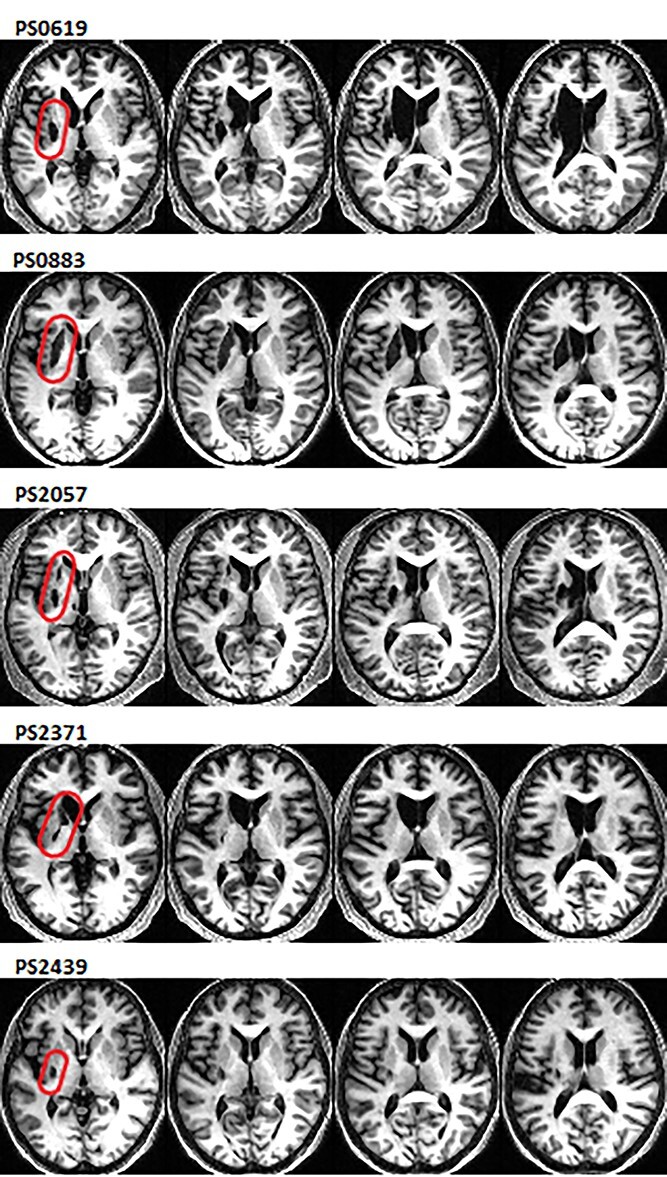
Lesions in POI with focal left dorsal striatal damage. Axial slices in MNI space showing lesions of the 5 POI, marked in red on the first slice. *Z* coordinates (from left to right) = 1, 7, 11, 17. Patient’s ID numbers from the PLORAS database are displayed above the images.

Among the 71 patients who completed the fMRI paradigm reported here (see below), we identified 5 patients of interest (POI) who had focal stroke damage to the left dorsal striatum, including the putamen. These patients were initially identified based on a neurologist’s description of the location and extent of the lesion, which is available for all the patients in our database. We then examined the structural MRI of the selected patients, to confirm that the lesion was indeed restricted to the left dorsal striatum. For 2 of these patients, the lesion affected only the left putamen, for 2 others the lesion extended from the putamen into the left caudate nucleus, and for the fifth patient the lesion affected the putamen and the surrounding white matter (Table 1 and Fig. 1). All 5 POI self-reported speech and language difficulties after their stroke but had largely recovered by the time they were tested with the CAT, more than a year later. Specifically, CAT scores were in the non-aphasic range for object naming, reading (words and nonwords) and repetition (words or nonwords) though one patient had a borderline score for word reading and another for nonword repetition (Supplementary Table 1).

**Fig. 2:**
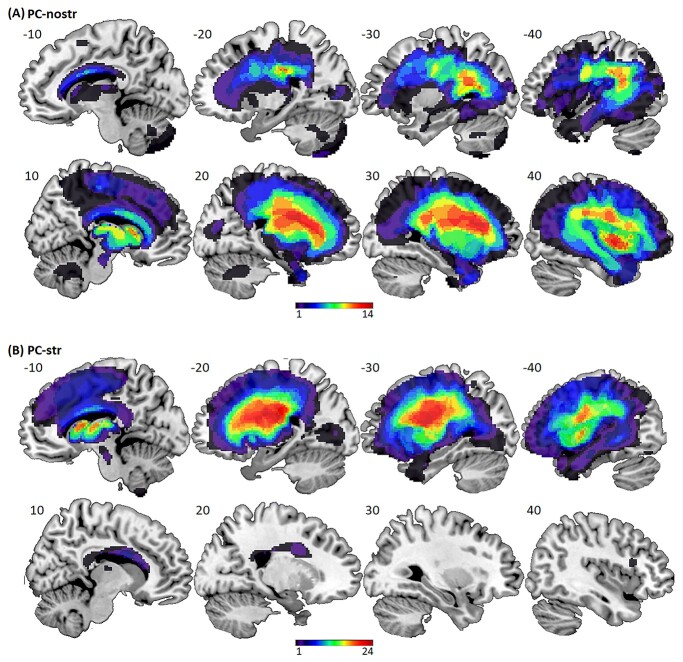
Lesion locations for the patient controls. Lesion overlap maps of binary lesion images generated by the automated lesion identification software, overlaid on sagittal slices in MNI space. (A) PC-nostr (*n* = 35); (B) PC-str (*n* = 31). Each panel displays the left hemisphere on top and the right hemisphere below it. Colors represent the number of patients with a lesion to each voxel, according to the axes below. *x* coordinates are displayed above the images.

All other patients who completed the fMRI study were assigned to one of 2 patient control groups: patient controls with no damage to the left dorsal striatum (PC-nostr, *n* = 35), and patient controls with damage to the left dorsal striatum (PC-str, *n* = 31), which extended to other gray or white matter structures distant to the left dorsal striatum, e.g. in parietal, frontal, and/or temporal regions; see Fig. 2 for lesion distribution. We included the PC-nostr group to test if abnormal activation seen in POI is lesion-site specific, and the PC-str group for testing if this activation can also be seen when the left dorsal striatal damage is not focal. The fourth group included NC (*n* = 54) who had no history of neurological or psychiatric illness. POI did not significantly differ from the control groups in the proportion of males and females (Chi square test, *P* > 0.05 for all) and did not differ from any of the control groups in age at scan (independent sample *t*-test, *P* > 0.05, though we note a trend for POI being older than NC, *t*_(57)_ = 1.90, *P* = 0.062). POI had significantly less years of formal education than the NC (independent sample *t*-test, *t*_(57)_ = 3.63, *P* = 0.001), but not compared with the patient control groups (independent sample *t*-test, *P* > 0.05 for both). Nor did they differ from the patient control groups in age at stroke or time since stroke (independent sample *t*-test, *P* > 0.05 for all). POI had significantly smaller lesions than patient controls (independent sample *t*-test, *t*_(35.0)_ = 4.46, *P* < 0.001 for comparing POI and PC-nostr, *t*_(32.6)_ = 4.32, *P* < 0.001 for comparing POI and PC-str) as quantified by an automated lesion identification software (Seghier et al. 2008; see section “Processing of structural MRI data” below for details). Summaries of demographic and clinical details for each group of participants are given in Table 2.

**Table 2:** Demographic and clinical details of the participant groups.

**Variable**		**POI (*n* = 5)**	**PC-nostr (*n* = 35)**	**PC-str (*n* = 31)**	**NC (*n* = 54)**
**Sex, *n***	Females/Males	2/3	10/25	14/17	33/21
**Age at fMRI (years)**	M (SD)	59 (10)	60 (12)	58 (9)	44 (18)
	Range	48–72	26–76	44–76	20–76
**Education (years)**	M (SD)	14.0 (2.1)	15.1 (3.2)	14.1 (2.6)	16.3 (1.3)
	Range	12–17	10–22	12–20	14–18
**Age at stroke onset (years)**	M (SD)	51 (16)	52 (12)	48 (12)	N/A
	Range	25–68	25–70	26–75	
**Time post-stroke at fMRI (years)**	M (SD)	8.8 (8.4)	7.5 (5.4)	9.5 (6.6)	N/A
	Range	1.9–22.9	0.9–21.7	0.9–23.9	
**Lesion volume (cm** ^**3**^ **)**	M (SD)	15.5 (17.2)	93.4 (92.7)	93.2 (90.4)	N/A
	Range	3.8–45.9	3.0–382.2	0^a^—372.0	

^a^0 = the automated lesion identification software did not segment the lesion (see section “Processing of structural MRI data”), and therefore, the lesion volume is defined as 0 cm^3^. M = mean.

All participants were native English speaking and right handed (according to the Edinburgh Inventory; Oldfield 1971), with normal or corrected-to-normal vision and hearing, and gave written informed consent to participate in the study, in accordance with the Declaration of Helsinki and the London Queen Square Research Ethics Committee (study codes: 13/LO/1515 and 19/LO/1755).

### In-scanner tasks

All participants performed 2 different experiments, combined in the same fMRI paradigm. Experiment 1 included 5 tasks, previously described by Sanjuán et al. (2015). Experiment 2 included 8 tasks, previously described by Oberhuber et al. (2016). Both experiments used a set of 120 objects, and pictures of these objects were realistically colored line drawings.

In Experiment 1, 3 tasks required overt speech production and 2 required a semantic decision, one with visual stimuli and a second with auditory stimuli. The semantic decision tasks were used as high-level baselines, which controlled for visual input (in visual semantic decision), auditory input (in auditory semantic decision), and object recognition (in both semantic decision tasks).

Each of the 5 tasks in Experiment 1 had 20 trials, and each trial presented 2 objects. To ensure that inter-patient variability could not be attributed to order differences, all participants performed the 5 tasks in the same following order:

(1) **Visual semantic decision:** Each trial comprised pictures of 2 non-interacting objects/animals that were either semantically related (e.g. cup and teapot) or unrelated (e.g. table and tortoise). Participants pressed the left button of a response box to semantically related items and the right button to indicate semantically unrelated items, using their index and middle fingers. Participants used their dominant hand unless unable to do so, in which case they used their non-dominant hand.
(2) **Naming 2 objects:** Participants viewed pictures of 2 objects/animals that were always semantically unrelated and non-interacting and named both objects using the phrase “x and y” (e.g. “bus and horse”).
(3) **Verb production**: Participants viewed pictures of 2 animals/objects interacting in one of 4 different ways (eating, drinking, jumping, and falling) and produced the infinitive form of the verb that describes the interaction. By way of illustration, we provide 4 examples. If the picture depicted a donkey eating a carrot the correct spoken response is “eating.” If the depiction showed bread falling out of a basket, the correct response would be “falling”; in the case of a king drinking from a cup, “drinking” would be correct and where a kangaroo is seen jumping over a car, “jumping” is the correct response.
(4) **Sentence production**: Participants saw pictures of 2 animals/objects interacting in 4 different ways (as in the verb production task) and produced a Subject–Verb–Object sentence (e.g. “The boy is jumping on the bed”).
(5) **Auditory semantic decision:** Participants heard the names of 2 objects that were either semantically related (e.g. dolphin and sea) or semantically unrelated (e.g. fridge and camera) and indicated if the items were semantically related or not using a button press, as in Task 1.

The stimuli were divided into 6 sets of 20 stimuli each (A, B, C, D, E, F). Task 1 presented A&B, Task 2 presented A&C, Task 3 presented B&D, Task 4 presented C&E, and Task 5 presented E&F. When a stimulus set was repeated, the objects were paired in a new way (e.g. zebra and lion on presentation 1 and zebra and fence on presentation 2). Sets D and F (40 objects) were not repeated.

Experiment 2 was included to see if results from Experiment 1 can be replicated in a different set of tasks, and to study the effects of various language factors on activation. It was conducted immediately after Experiment 1 and included 8 overt speech production tasks, with 40 trials each, that were designed to vary factorially the demands on verbal input processing (words and pseudowords vs. object pictures/sounds or meaningless patterns), semantic input processing (words and object pictures/sounds vs. pseudowords and meaningless patterns), and stimulus modality (visual vs. auditory). Tasks were presented in the following order:

(1) **Reading aloud familiar animal or object names**, using stimulus sets A and D (presented as pictures in Experiment 1).
(2) **Repeating heard familiar animal or object names**, using stimulus sets B and D (previously presented as pictures or written words).
(3) **Naming familiar animals or objects from pictures**, using sets F (previously presented as auditory object names) and E (previously presented as auditory object names and pictures).
(4) **Naming the color** of meaningless patterns, created by scrambling both global and local features of the object pictures, which were then manually edited to accentuate one of 5 colors (i.e. blue, orange, red, yellow, and green) with 8 repetitions of each color.
(5) **Naming familiar animals or objects from their sounds**, taken from the NESSTI sound library (Hocking et al. 2013). This task presented 3 stimuli from each of the sets B, C, and F, 2 from set A, and 1 from set D, along with 8 new objects. The participants were familiarized with all these object sounds prior to the experiment to ensure that they were easily recognizable.
(6) **Reading aloud pseudowords,** created using a nonword generator (Duyck et al. 2004) that matched the pseudowords to the real words used in the word reading/repetition tasks, for bigram frequency, number of orthographic neighbors, and spoken word length (for details see Oberhuber et al. 2016).
(7) **Repeating heard pseudowords** from the same nonword generator.
(8) Saying whether a voice producing humming sounds, with no semantic or verbal input (**auditory humming**), was most likely to be “male” or “female.”

### Procedure

Prior to scanning, each participant was trained on all tasks, except the sound naming task, using different stimuli from those presented in the scanner. For the sound naming task (task 5 in Experiment 2), we used the same stimuli in the practice and scanning sessions because pilot testing showed that neurotypical participants required more practice on this condition to achieve highly accurate and consistent object recognition. To reduce repetition effects, the stimuli for the sound naming task were presented in a different order during fMRI data acquisition than during the practice session.

Each task was executed during its own scanning run with 4 blocks of trials alternating with 16 s of resting while fixating on a central cross. Scanning started with the instructions “Get ready” displayed on the screen during which 5 dummy scans were acquired. Each of the 4 blocks was preceded by a displayed instruction to prepare for the next condition (e.g. “Repeat”), lasting for the length of 1 repetition time (TR = 3080 ms), and followed by 16 s of rest.

In Experiment 1, each of the 4 blocks had 5 trials with each trial presenting 2 objects. Trials were presented at a rate of one trial per 5 s with 2.5 s stimulus duration (followed by 2.5 s rest) in the visual modality, and 1.76–2.5 s stimulus duration (followed by 2.5–3.24 s rest) in the auditory modality.

In Experiment 2, each of the 4 blocks had 10 trials presented at a rate of one trial every 2.5 s. Visual stimuli remained on the screen for 1.5 s followed by 1 s fixation, and the mean durations for presentation of auditory stimuli were: 0.63, 0.65, 1.45, and 1.05 s for words, pseudowords, object sounds, and humming, respectively.

Data acquisition per participant for both experiments combined lasted on average 90 min including setting up, getting the participant into the scanner, and structural and functional imaging.

Stimuli were presented using COGENT (http://www.vislab.ucl.ac.uk/cogent.php) and run in MATLAB 2010a (MathWorks, Sherbon, MA). During all conditions, participants were asked to keep their body and head as still as possible and their eyes open with fixation on the cross at the center of the screen. Visual stimuli were presented using an LCD projector on a screen placed at the head-end of the scanner bore and an adjustable mirror placed on the head coil to allow participants’ viewing of the screen. Pictures subtended a visual angle of 7.4 degrees, with a screen resolution of 1024 × 768 (after scaling to 350 × 350 pixels). Words and pseudowords were presented in lower case Helvetica. Their visual angle ranged from 1.47 to 4.41 degrees with the majority of words (with 5 letters) extending 1.84–2.2 degrees. Auditory stimuli were presented using headphones designed to filter in-scanner noise (MR Confon, Magdeburg, Germany). Volume was adjusted for each participant to maximize audibility during a practice task of single word repetition, in which it was confirmed that participants could hear the stimuli over the scanner noise.

### In-scanner behavioral data

#### Behavioral response acquisition

For the semantic decision tasks, response time and type were recorded from a button press using an MRI compatible button box. In all other tasks, overt spoken responses were recorded using a noise-canceling MRI compatible microphone (FOMRI III™ Optoacoustics, Or-Yehuda, Israel). They were transcribed at the time of imaging and scored for accuracy off-line by listening to the audio files. Each response was categorized as correct (e.g. both “fridge” and “refrigerator” were accepted as correct naming responses for a picture of a fridge) or incorrect (when the response did not match the target and/or was delayed).

Reaction times (RTs) for spoken responses were obtained from the audio files using an adaptive moving window filter that was tailored to each participant. The optimal window length (i.e. the width which maximally smoothed the audio stream) was based on a portion of the audio file collected during the resting baseline between blocks of stimuli. After smoothing the whole time series, we defined the onset of speech as a rise in the absolute amplitude of the smoothed audio stream beyond 3 SDs from the mean. We analyzed the average RT per condition per participant, for correct trials only. Due to technical issues, we missed 10 data points (data point = RT averaged over trials within one of the 13 tasks) corresponding to 0.6% of the 1625 data points acquired (13 tasks × 125 participants). One data point was missing from each of 3 NC and 5 patient controls and 2 data points were missing from a further, fourth NC (10 data points in total).

#### Analysis of behavioral performance

The in-scanner behavioral responses were analyzed using IBM SPSS Statistics for Windows (version 27.0, IBM Corp., Armonk, NY).

To examine the relation between performance (accuracy and RT) and demographic (education, age at scan) or stroke-related variables (time between stroke and scan, lesion volume), we used 2-tailed Spearman’s rho, with Benjamini–Hochberg False Discovery Rate (FDR) correction for multiple comparisons. Correlations are reported for the entire cohort of participants (*n* = 125), and also separately for the NC (*n* = 54) and the full patient cohort (*n* = 71). We then tested whether accuracy scores and RTs show group differences and interactions with task parameters, separately in the 2 experiments. As the 2 patient control groups (PC-nostr and PC-str) did not differ in either accuracy or response time on any of the 13 tasks, the data of the 2 groups was combined (PC) for all behavioral analyses.

Experiment 1: Using independent Multivariate analysis of variances (MANOVAs), we tested for the main effect of group (POI, PC, NC) and task (3 × 3 MANOVA for the 3 speech production tasks: naming 2 objects, verb production, and sentence production; and a 3 × 2 MANOVA for the 2 semantic decision tasks: visual and auditory).

Experiment 2: Using a factorial design, and 3 × 2 × 2 × 2 MANOVAs, we tested for the main effects of group (POI, PC, NC), verbal input, semantic input, and stimulus modality, together with their interactions.

Post hoc tests were conducted using independent sample 2-tailed *t*-tests. We did not compare RT between presentation modalities in either experiment, as stimulus presentation time could not be matched for the auditory and visual conditions, and because in the semantic decision tasks of Experiment 1, some patients used their nondominant hand (because of stroke-related difficulties with their dominant hand), making their response potentially slower.

### Acquisition of MRI data

We used a 3 T Trio scanner (Siemens Healthcare, Erlangen, Germany) to acquire all images. An optimized 3D modified driven equilibrium Fourier transform sequence was used to acquire the anatomical high-resolution T1-weighted structural images with a voxel size of 1 × 1 × 1 mm (TR/echo time [TE]/inversion time = 7.92/ 2.48/910 msec; flip angle = 7°, matrix size = 256 × 224, 176 sagittal slices).

Functional images were acquired using a 12-channel head coil and a gradient-echo EPI sequence with 3 × 3 mm in-plane resolution (TR/TE = 3080/30 msec, flip angle = 90°, field of view = 192 mm, matrix size = 64 × 64, slice thickness = 2 mm, interslice gap = 1 mm, 44 axial slices). 66 image volumes per session (i.e. task) were acquired, including 5 dummy scans to allow for magnetization to reach equilibrium. The TR was chosen to maximize whole-brain coverage and to ensure that slice acquisition onset was offset with stimulus onset, which allowed for distributed sampling of slice acquisition across the study (Veltman et al. 2002).

### Processing of structural MRI data

The T1-weighted anatomical whole-brain volume of each participant was analyzed with an automated lesion identification toolbox (Seghier et al. 2008). The toolbox is a modification of the unified segmentation–normalization routine implemented in SPM8, which has been shown to be more accurate and robust compared with other methods, when dealing with lesioned brains (Crinion et al. 2007). The toolbox converts a scanner-sensitive raw image into a quantitative assessment of structural abnormality by first segmenting the whole brain into 4 tissue classes: gray matter, white matter, and cerebrospinal fluid (as used in the standard routine), with the addition of a fourth (atypical tissue) class. This fourth class represents outlier voxels within gray and white matter that are far from the normal range of the voxel values in NC. The tissue affected by a lesion will therefore be identified as an outlier and classified as atypical. The 4 generated tissue priors are then used in the next segmentation run, which repeats the same process again. This ensures that the normalization procedure weights the abnormal tissue appropriately and helps to avoid misclassification of damaged voxels. The output is a binary image that delineates the lesion(s) and here was used to estimate lesion volume and visualize the lesions of the patient controls. The software does not delineate lesions <1 cm^3^ (the default automated setting), and therefore did not generate a binary lesion image for 3 patients who had multiple small lacunae. In order to maintain consistent lesion volume calculation across the entire cohort, the lesion volumes for these 3 patients were entered as 0. All automatically generated lesion images were inspected by eye, and compared with the lesion description reported by a neurologist.

### Pre-processing and first level modeling of functional MRI data

Data pre-processing was performed in the Statistical Parametric Mapping software (SPM12; Wellcome Centre for Human Neuroimaging, London, UK; https://www.fil.ion.ucl.ac.uk/spm/), running in MATLAB environment (2021a Mathworks, Sherbon, MA). Functional volumes were spatially realigned to the first EPI volume and unwarped to compensate for nonlinear distortions caused by head movement or magnetic field inhomogeneity. We used the unwarping procedure in which the interaction between head movement and any inhomogeneity in the T2^*^ signal is modeled. To spatially normalize all realigned EPI scans to the MNI standard space, we co-registered the mean EPI image to the anatomical T1 image, spatially normalized the anatomical image using the new unified segmentation–normalization routine and applied the deformation field parameters to the EPI images. The original resolution of the images was maintained during normalization. After the normalization procedure, the functional images were spatially smoothed with a 6-mm full-width half-maximum isotropic Gaussian kernel to compensate for residual anatomical variability and to permit application of Gaussian random-field theory for statistical inference (Friston et al. 1995). Each preprocessed functional volume was individually inspected for oddities before statistical analyses.

For the first level analysis of each participant, data from each task were entered into a subject-specific fixed-effect analysis using the general linear model (Friston et al. 1995). Stimulus functions were convolved with a canonical hemodynamic response function. To exclude low-frequency confounds, the data were high-pass filtered using a set of discrete cosine basis functions with a cut-off period of 128 s. We maximized brain coverage by including all voxels whose mean value is at least 20% of the global signal. All stimulus onset times were modeled as single events. Correct, incorrect, and no responses were modeled separately, and the results focus only on activation observed during correct responses.

For each subject-specific first level analysis, we compared activation for correct trials to rest, for each task (5 tasks in Experiment 1 and 8 tasks in Experiment 2). As some participants had low performance on specific tasks, allowing for only a limited number of trials to be included in the first level contrasts (Task > Rest), the activation signal might be less robustly estimated in the patients than the NC. This might result in more error variance in the patient group and less significant activation. We addressed this in 2 ways. First, we computed the main effect of speech production compared with rest; i.e. (Tasks 2, 3, and 4 > Rest) in Experiment 1 and (all 8 speech production tasks > Rest) in Experiment 2. This ensured that multiple correct trial responses contributed to the effect. For example, although POI PS0619 made only 55% correct responses during the auditory object naming task (Experiment 2), accuracy rose to 83% correct (267/320 trials) across all 8 speech production tasks, providing a high number of trials to estimate the main effect of speech production. Second, we evaluated whether activation was proportional to the number of trials entered into the contrast (which was equivalent to task accuracy). No contrasts, which combined tasks from the 2 different experiments, were computed.

To examine neural activation within the dorsal striatum, in each of the 5 POI with focal damage to this region, we focused on activation within an anatomical mask that included the bilateral putamen and caudate nucleus, derived from IBASPM (Alemán-Gómez et al. 2006) implemented in Pickatlas (Maldjian et al. 2003). The statistical threshold was set at *P* < 0.001 uncorrected.

### Second level analysis of functional MRI

For the main analyses of interest (POI > NC; POI < NC) we only report results which are significant at a standard voxel-level threshold of *P* < 0.05 family-wise-error (FWE) corrected for multiple comparisons across the whole brain. For the follow-up (post hoc) ROI analyses, we report results at a more lenient voxel-level threshold of *P* < 0.001 uncorrected, in order to (i) avoid misrepresenting null results when trends are found and (ii) apply small volume correction when the contrast for the post hoc test was orthogonal to the contrast generating the ROI. When results are not significant at a corrected threshold level, we also present the activation of individual participants (in addition to the group result) and analyze the inter-patient variability in activation, see details below.

#### Dorsal striatal activation in each patient of interest, during speech production

Separate second level analyses were conducted for each of the 5 POI. In each analysis, the patient was modeled as one group and the 54 NC were modeled as a second group. The data were the first level speech production contrasts from either Experiment 1 or Experiment 2 (10 different analyses in total). We tested for group differences (NC > each POI) within the anatomical mask, described above, that included the bilateral putamen and caudate nucleus, derived from IBASPM (Alemán-Gómez et al. 2006). The statistical threshold was set at *P* < 0.001 uncorrected.

#### The effect of dorsal striatal damage on whole-brain activation during speech production

The second level analysis of Experiment 1 included 4 groups (POI, NC, PC-nostr, PC-str) and 5 tasks, with 2 covariates (age and education that were not perfectly matched across group). From this analysis, we identified ROIs that were more or less activated (voxel-level statistical threshold of *P* < 0.05 FWE-corrected for multiple comparisons across the whole brain) for POI than NC for speech production (across 3 tasks) compared with both rest and semantic decision (across 2 tasks). This resulted in 4 contrasts: (POI > NC) and (NC > POI), for (3 speech production tasks > Rest) and (3 speech production tasks > 2 semantic tasks).

Regions showing higher or lower activation for POI than NC, during speech production compared with rest, and compared with semantic processing, became our ROI for further analyses.

A separate second level analysis was conducted for Experiment 2, with 4 groups (POI, NC, PC-nostr, PC-str) and the 8 speech production tasks. Focusing on the ROI from Experiment 1, we report group differences (i.e. POI vs. NC, PC-nostr, or PC-str) for the main effect of speech production (across conditions) compared with rest and the interactions between group and (i) verbal versus nonverbal input; (ii) semantic versus nonsemantic input; and (iii) stimulus modality (visual vs. auditory).

#### Normal function of the ROIs

To examine whether activation in the ROIs is sensitive to the differing demands of verbal input, semantic input, stimulus modality, sentence processing, and articulation in the intact brain, we conducted a second level analysis with one group (NC), 13 tasks (5 tasks from Experiment 1, 8 tasks from Experiment 2), no covariates, and a voxel-level statistical threshold of *P* < 0.05 FWE-corrected for multiple comparisons across the whole brain. In Experiment 1 the following contrasts were examined: (i) speech production > semantic tasks (tasks 2, 3, 4 > tasks 1 and 5); (ii) sentence production > naming 2 objects (task 4 > task 2); (iii) naming 2 objects > verb production (task 2 > task 3); and, (iv) verb production > semantic decision (task 3 > tasks 1 and 5). In analyzing the data of Experiment 2 we used the full 2 × 2 × 2 factorial analysis.

### Post hoc analysis of functional MRI data

#### Inter-patient variability in ROI activation

We describe the occurrence of enhanced activation (compared with NC) in each of the patient groups (POI, PC-nostr, PC-str) in Experiment 1 and 2. To examine whether ROI activation values in patients were related to (i) lesion volume; (ii) time post-stroke; and, (iii) performance (accuracy and RT); we used 2-tailed Spearman’s rho tests and Benjamini–Hochberg FDR correction for multiple comparisons. Activation values were defined as the task-specific average principal eigenvariate within a 3-mm radius sphere centered on the peak coordinates of the ROI and were extracted using the eigenvariate function in SPM12, and averaged across the relevant conditions (e.g. verbal input, semantic input).

#### The effect of performance on activation in NC

For the first analysis, NC were divided into 2 groups based on their accuracy level across tasks (above or below the mean group accuracy), and for the second analysis, based on their RT across tasks (faster or slower than mean group RT). Using 2 independent 2 × 2 × 2 MANOVAs, we tested for the effects of Group (High vs. Low Accuracy or Fast vs. Low RT), Experiment (Experiment 1 vs. Experiment 2), and Region, on activation during speech production. Post hoc tests were conducted using independent sample 2-tailed *t*-tests. Activation was defined as above.

## Results

### Behavioral performance inside the scanner

Across the entire cohort of participants, higher accuracy was associated with faster RTs on correct trials, in both experiments. In addition, older age and fewer years of formal education were associated with lower accuracy and slower response times. Among the entire cohort of patients, larger lesions correlated with lower accuracy and slower responses in Experiment 1, but not in Experiment 2. There were no significant correlations between time post-stroke and performance in either experiment (Spearman’s rho, *P* < 0.05 FDR-corrected for all significant results, see Supplementary Table 2 for further details). Correlations within the POI group were not calculated due to sample size, however, in all significant pairwise correlations, individual values for all 5 POI fell within the 95% confidence interval of the correlation observed in the patient controls.

Performance in most tasks, in both Experiment 1 and 2, was best (highest accuracy, lowest response times) for NC and worst for PC (Supplementary Figs. 1 and 2). POI accuracy did not differ from NC accuracy in either Experiment 1 or 2, but POI were slower during Experiment 1, not Experiment 2. For complete MANOVAs and post hoc *t*-tests results see Supplementary Material Sections 2.1 and 2.2.

### Dorsal striatal activation in each patient of interest during speech production

**Fig. 3:**
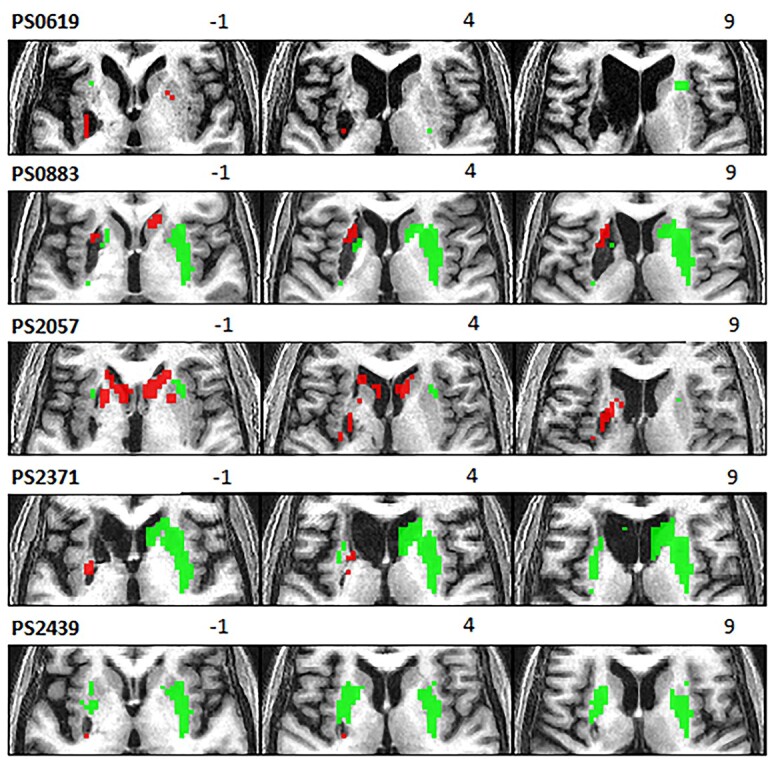
Dorsal striatal activation in POI in experiment 1. Thresholded activation during speech production in Experiment 1, displayed within the boundaries of the dorsal striatum bilaterally. Activation (Green) is derived from each participant’s first level analysis, for the contrast (3 speech production tasks > rest). Underactivation (red) is derived from second level analyses, for the contrast (NC group > each patient). Activations are presented at a voxel-level statistical threshold of *P* < 0.05 FWE-corrected across the whole brain, other than: (PS2057 tasks > rest) and (NC > PS2371), which are displayed at a voxel-level statistical threshold of *P* < 0.001 uncorrected. Patient’s ID numbers from the PLORAS database and *z* coordinates are displayed above the images.

Individual analyses of data for each POI showed significant activation in undamaged parts of the dorsal striatum during speech production, with, as expected, significantly lower activation than NC, particularly within the boundaries of each patient’s lesion (see Fig. 3 for Experiment 1 activation). When the POI were treated as a group, the difference between dorsal striatum activation in POI and NC did not reach significance (*P* > 0.001 uncorrected) due to inter-patient differences in lesion location/extent and the precise locus of activation in undamaged parts of the dorsal striatum. Similar results were found in Experiment 2, see Supplementary Fig. 3.

### The effect of dorsal striatum damage on whole-brain activation during speech production

**Fig. 4:**
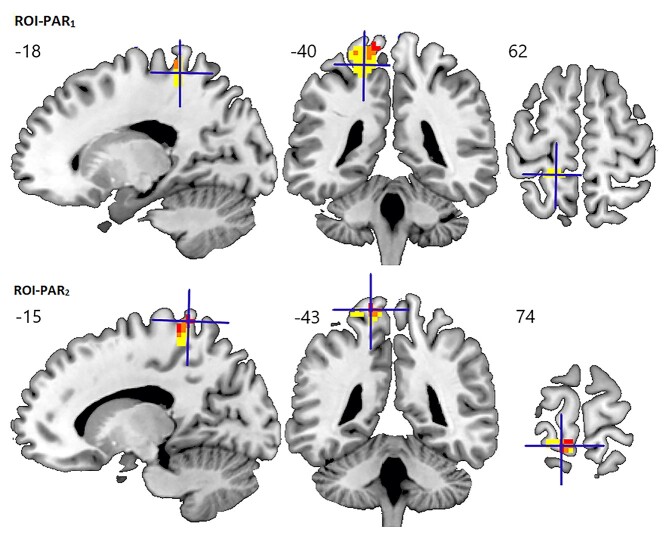
Areas of enhanced activation in the left superior parietal cortex. ROI-PAR_1_ (POI > NC, for 3 speech production tasks > 2 semantic decision tasks) is shown in yellow, and ROI-PAR_2_ (POI > NC, for verbal > nonverbal input) is shown in red. The overlap between the 2 ROIs is shown in orange. The peak coordinate of each ROI (ROI-PAR_1_ in the top panel, ROI-PAR_2_ in the bottom panel) is marked by a cross on sagittal (left), coronal (middle), and axial (right) slices in MNI space. Clusters of activation are displayed at a voxel-level statistical threshold of *P* < 0.001 uncorrected. MNI coordinates are displayed above the images.

**Fig. 5:**
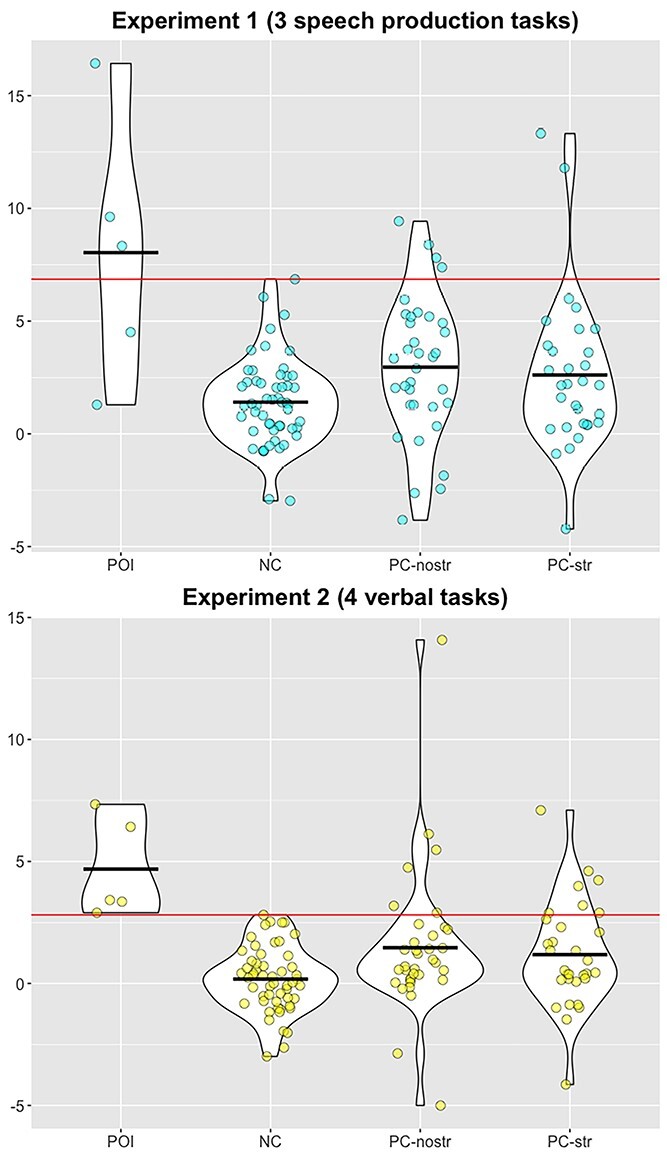
Inter-subject variability in the parietal ROI for speech production tasks. Individual activation in Experiment 1 (ROI-PAR_1_, top) and Experiment 2 (ROI-PAR_2_, bottom) within the POI, NC, PC-nostr, and PC-str groups. y-axis represents the mean of the principal eigenvariate within a 3-mm radius sphere centred on the peak coordinate (Table 3), and averaged across the relevant tasks. Black lines represent the group averages. Red line represents the maximum value in the NC group.

In Experiment 1, we found no clusters where activation was significantly reduced in the POI group compared with the NC group. However, POI had higher activation than NC (*P* < 0.05 FWE-corrected) and PC (*P* < 0.001 uncorrected) during speech production compared with both rest and semantic decision, in the medial part of the left superior parietal cortex (ROI-PAR_1_ Exp. 1 in Table 3). This region included the somatosensory cortex in the post-central gyrus and cingulate gyrus, Brodmann Areas 1–3 and 5 (Figs. 4 and 5) and became our ROI for further analyses.

**Table 3:** Left superior parietal regions of enhanced activation among POI.

**Region**	**Condition**	**Peak coordinates**			**Cluster size**	**Z-score**			
		**x**	**y**	**z**		**POI > NC**	**POI > PC-nostr**	**POI > PC-str**	**PC > NC**
ROI-PAR_1_ (Exp. 1)	3 speech production tasks > Rest	−18	−40	62	53	5.09	3.90	4.19	ns
	3 speech production > 2 semantic tasks				64	5.56	4.51	4.43	3.37
ROI-PAR_1_ (Exp. 2)	8 speech production tasks > Rest	−15	−40	74	2	3.22^§^	ns	ns	ns
	Verbal input > Rest	−12	−43	74	25	4.76	3.61	3.80	ns
	Verbal > Nonverbal input				16	4.97	4.29	4.78^*^	ns
ROI-PAR_2_ (Exp. 2)	Verbal input > Rest	−12	−40	74	101	4.99	3.83	4.09	ns
	Verbal > Nonverbal input	−15	−43	74	25	5.04	4.39	4.87^*^	ns

Anatomical and statistical details of the left superior parietal ROI where activation for speech production compared with semantic decision was significantly greater for POI than NC in Experiment 1 (ROI-PAR_1_, Exp. 1). The peak left superior parietal activation in Experiment 2 (verbal input tasks, Exp. 2) in a whole-brain analysis (ROI-PAR_2_) partially overlapped with ROI-PAR_1_ (Fig. 4). All Z-scores for POI > NC are significant at a voxel-level threshold of *P* < 0.05 FWE-corrected for multiple comparisons across the whole brain, unless marked (§). For POI > PC and PC > NC, Z-scores are significant at a voxel-level threshold of *P* < 0.001 uncorrected, or *P* < 0.05 FWE-corrected for multiple comparisons across the whole brain (when marked with an asterisk). Note that POI > PC effects cannot be explained by damage to the ROI in the PC groups because only one of the 66 PC participants had damage to the ROI. Peak coordinates are in MNI space; cluster size in number of voxels is reported with a voxel-level statistical threshold of *P* < 0.001 uncorrected.

**Fig. 6:**
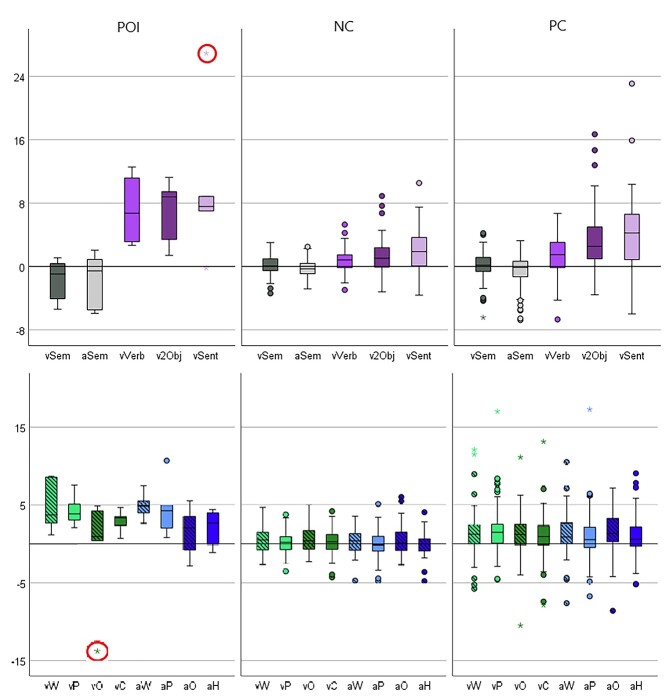
Task-dependent activations in the parietal ROI. Activation (*y* axis) for each group of participants (POI, NC, PC) in ROI-PAR_1_ for Experiment 1 (top) and ROI-PAR_2_ for Experiment 2 (bottom). As PC-nostr and PC-str did not differ in activation in 10 of the 11 overt speech production tasks (exception being PC-nostr > PC-str for verb production), their data were combined (PC). Activation = the mean of the principal eigenvariate within a 3 mm radius sphere centered on the peak coordinates reported in Table 3. The box represents the interquartile range (IQR), and the hinges represent the minimum and maximum values (excluding outliers). Stars represent outliers, defined as values that are > 3 times the IQR either above Q3 or below Q1. Full circles represent values which are >1.5 times the IQR. Encircled in red are task specific outliers, see Supplementary Material Section 4. Experiment 1 tasks from left to right are: visual semantic decision (vSem); auditory semantic decision (aSem); naming 2 visual objects (v2Obj); verb production (vVerb); sentence production (vSent). The speech production tasks are purple and the semantic decision tasks are gray. Experiment 2 tasks from left to right are: visual word reading (vW); visual pseudoword reading (vP); visual object naming (vO); visual color naming (vC); auditory word repetition (aW); auditory pseudoword repetition (aP); auditory object naming (aO); auditory humming (aH). Green = visual presentation, blue = auditory presentation, light colors = verbal input, dark colors = nonverbal input, stripes = semantic input, no stripes = nonsemantic input.

In Experiment 2, the superior parietal ROI was not significantly more activated in POI than NC during successful speech production (8 tasks > Rest). However, it was significantly more activated for POI than NC (*P* < 0.05 FWE-corrected) during tasks with verbal input, for the contrasts: (Verbal input > Rest) and (Verbal > Nonverbal input), with a corresponding trend for POI compared with PC (*P* < 0.001 uncorrected; see ROI-PAR_1_ Exp. 2 in Table 3, and Figs. 5 and 6).

This significant interaction between POI > NC and the verbal versus nonverbal input conditions is particularly interesting because, in NC, the effect of verbal > nonverbal input conditions was not significant in the parietal ROI but was significant in the left dorsal striatum (Table 4) in a cluster spanning the anterior and posterior putamen (*P* < 0.05 FWE-corrected). The interactions between Group (POI vs. NC) and (i) Semantic input, or (ii) Stimulus modality were not significant (*P* > 0.05 FWE-corrected across the whole brain).

**Table 4:** Activation in ROIs among NC.

**Region**	**Condition**	**Peak coordinates**			**Cluster size**	**z-score**
		***x***	***y***	***z***		
Experiment 1						
ROI-PAR_1_	Speech production > Semantic decision	−18	−37	62	64	7.50
	Sentence production > Naming 2 objects	−27	−40	68	11	4.02^*^
	Naming 2 objects > Verb production	−18	−37	56	25	5.32
	Verb production > Semantic decision	−18	−37	65	34	5.29
ROI-dSTR	Speech production > Semantic decision	−30	−13	−4	392	7.75
	Sentence production > Naming 2 objects	−21	14	−4	9	4.12^*^
	Naming 2 objects > Verb production	−21	17	8	160	6.98
	Verb production > Semantic decision	−18	−22	20	80	5.46
		−30	−10	−7	23	5.07
Experiment 2						
ROI-PAR_2_	8 speech production tasks > Rest	−15	−37	65	6	5.23
ROI-dSTR	8 speech production tasks > Rest	−21	−1	11	454	8.67
	Verbal input > Rest	−21	5	5	448	8.74
	Verbal > Nonverbal input	−21	5	2	139	6.26

Statistical details of NC activation in left superior parietal cortex (ROI-PAR_1_), and left dorsal striatum (ROI-dSTR) in Experiments 1 and 2 (Exp. 1, Exp. 2). All Z-scores are significant at a voxel-level threshold of *P* < 0.05 FWE-corrected for multiple comparisons across the whole brain, except those marked with an asterisk, which were significant at *P* < 0.001 uncorrected. Peak coordinates are in MNI space; cluster size in number of voxels is reported with a voxel-level statistical threshold of *P* < 0.001 uncorrected.

### Normal function of left superior parietal ROI

In the NC group, the parietal ROI was significantly more activated (*P* < 0.05 FWE-corrected across the whole brain) during speech production compared with semantic decision or rest in Experiment 1, and across all 8 speech production tasks compared with rest in Experiment 2 (Fig. 6). In Experiment 1, activation in the parietal ROI also increased with the demands on speech articulation, as measured by the number of words that are required in each response. Specifically, greater activation was observed (*P* < 0.05 FWE-corrected across the whole brain) for: (Naming 2 objects [Task 2] > Verb production [Task 3]), and (Verb production [Task 3] > Semantic decisions [Tasks 1 & 5], which do not require overt articulation); see Table 4 and Fig. 6. There was a strong trend for higher activation in the parietal ROI for (Sentence production [Task 4] > Naming 2 objects [Task 2]) at *P* < 0.001 uncorrected. The same pattern of activation was found in the left dorsal striatum (ROI-dSTR; Table 4).

### Inter-patient variability in left superior parietal ROI activation

At an individual patient level (Fig. 5), all POI showed ROI activation greater than the maximum activation for NC during reading and repetition (the verbal input tasks in Experiment 2) but, for 2 POI, activation was within the (high) normal range during the speech production tasks in Experiment 1.

In control patients (PC), the incidence of enhanced activation in the parietal ROI (i.e. greater than the maximum activation for NC) was not significantly different in PC-str and PC-nostr (Chi square test, *P* > 0.05), as enhanced activation in the parietal ROI was observed in 2/31 (6.5%) PC-str and 4/35 (11.4%) PC-nostr in Experiment 1, and 7/31 (22.6%) PC-str and 6/35 (17.1%) PC-nostr in Experiment 2. Among the 2 PC-str participants showing enhanced activation in Experiment 1, only one also showed enhanced activation in Experiment 2. Similarly, among the 4 PC-nostr participants showing enhanced activation in Experiment 1, 3 showed enhanced activation also in Experiment 2.

In addition, variability in parietal ROI activation among patients could not be explained by time post-stroke, lesion volume (whole-brain or only left hemisphere), or performance (accuracy or RT; Spearman’s rho, *P* > 0.05 for all).

### The effect of performance variables on ROI activation in NC

Most interestingly, comparing activation levels between neurotypical participants with high and low accuracy scores (i.e. above or below the mean group accuracy) across seven speech production tasks (3 tasks from Experiment 1 and 4 verbal input tasks from Experiment 2), revealed a significant main effect of Experiment (Experiment 1 vs. Experiment 2, *F*_(52,1)_ = 42.6, *P* < 0.001), and Region (ROI-PAR vs. ROI-dSTR, *F*_(52,1)_ = 40.1, *P* < 0.001). And while there was no significant effect of Accuracy Group (*F*_(52,1)_ = 2.6, *P* = 0.114), there was a significant interaction between Accuracy Group and Experiment (*F*_(52,1)_ = 15.7, *P* < 0.001) and a trend for interaction between Accuracy Group and ROI (*F*_(52,1)_ = 3.9, *P* = 0.053). Post hoc tests showed that in Experiment 1, neurotypical participants with lower accuracy had significantly higher activation in the parietal region (*t*_(52)_ = 2.9, *P* = 0.006) and a trend in the same direction in the dorsal striatal region (*t*_(52)_ = 1.9, *P* = 0.052). In Experiment 2, there was no significant difference in parietal activation between the lower and higher accuracy groups (*t*_(52)_ = 0.02, *P* = 0.986), but dorsal striatal activation was significantly higher in participants with higher accuracy (*t*_(38.4)_ = 2.12, *P* = 0.041). See Supplementary Fig. 4.

When participants were divided into 2 groups based on average RT (i.e. faster or slower than mean group RT), there was no significant effect of RT Group on activation, and no significant interactions between RT Group and Experiment or Region (*P* > 0.05 for all). See Supplementary Fig. 4.

Lastly, for sentence production, there was a sufficient number of incorrect trials for us to test whether the left superior parietal ROIs were more activated for correct than incorrect trials. Focusing only on participants who made >1 sentence production error, we found significantly higher activation for correct > incorrect trials in NC (*n* = 42, ROI-PAR_1_ Exp. 1 and ROI-PAR_2_ Exp. 2), PC (*n* = 63, ROI-PAR_1_ Exp. 1), and POI (*n* = 5, ROI-PAR_1_ Exp. 1 and ROI-PAR_2_ Exp. 2) using a voxel threshold *P* < 0.05 FWE-corrected across the ROI, see Supplementary Section 6 for further details.

## Discussion

In the neurotypical brain, left dorsal striatal activation contributes to speech production (Jacquemot and Bachoud-Lévi 2021). The goal of this study was to identify the neural mechanisms that support successful speech production in patients with focal left dorsal striatal damage affecting the putamen (POI). Compared with the activation patterns of a control group of neurotypical participants (NC), POI showed higher activation during correct overt spoken responses in a left superior parietal region that included the dorsal portion of the somatosensory cortex, and parts of the cingulate gyrus (Experiment 1) and precuneus (Experiment 2). Enhanced left superior parietal activation was also seen among some of the patient controls regardless of lesion location (i.e. in both PC-str and PC-nostr), which suggests that upregulation in this region is not lesion-site specific but rather is associated with the effort required to successfully perform the speech production tasks. Data from the NC support this hypothesis, as in this participant group, left superior parietal activation for correct spoken responses increased (i) with the demands on articulation, and (ii) in participants who made more errors (Experiment 1). Below, we consider the functional contribution of the parietal ROI in relation to the left dorsal striatum and the implications for understanding normal and post-stroke speech production.

### Understanding the function of the left superior parietal cortex and left dorsal striatum during correct spoken responses

Our data from neurotypical participants show that activation in the left superior parietal ROI and the left dorsal striatum increase with the demands on articulation (Experiment 1). Most interestingly, neurotypical participants who are more error-prone, also had enhanced activation, in both of these regions, during accurate speech production in Experiment 1. This suggests that the left superior parietal ROI and the left dorsal striatum are working harder to sustain successful speech production in participants who are error-prone.

The findings from Experiment 2 provide two sources of tentative evidence that the left parietal ROI may help to compensate for loss of function when the left dorsal striatum is damaged. First, neurotypical participants who were more error-prone had lower left dorsal striatal activation in Experiment 2 despite higher left superior parietal activation in Experiment 1. Second, activation for speech production in response to verbal (reading and repeating) compared with nonverbal stimuli (pictures, colors, sounds, and humming) was greater in the left dorsal striatal for NC, but in the left parietal ROI for patients (who had reduced activation in the left dorsal striatum).

The enhanced parietal responses in Experiment 1 but not in Experiment 2 may be explained by (i) our previous finding (Ekert et al. 2021) that object naming activation is higher when 2 objects are presented per stimulus compared with when only 1 object is presented per stimulus, even when the total number of objects is controlled, and (ii) the study design, as our participants were presented with the same object concepts in Experiments 1 and 2, and these concepts were therefore less novel in Experiment 2. Higher parietal activation may therefore be related to the naming task in Experiment 1 being more challenging.

Experimental data from different domains allow for further refinement. In a previous study, activation in the vicinity of our parietal ROI (peak coordinate: *x* = −16, *y* = −44, *z* = 64) was found when multilinguals were engaged in simultaneous interpretation (i.e. translating into one language while listening to a different language), an especially taxing linguistic task requiring both input processing and overt language production (Hervais-Adelman et al. 2015). Parietal activation in this study was also associated with the overlap of speech input and overt interpretation suggesting it is especially required for moment-to-moment control and response initiation. In a second study, parietal activation in the same vicinity was observed (peak coordinate *x* = −20, *y* = −42, *z* = 62) when participants comprehended speech in noise, over merely hearing but not understanding it (Bishop and Miller 2009). In both these studies, the effects were also seen in the bilateral putamen and caudate nuclei. One plausible, but untested, hypothesis is that dorsal striatal activation is related to online language control, whereas parietal activation is linked to increased attention during high-level language monitoring (Bishop and Miller 2009; Hervais-Adelman et al. 2015).

Pulling these results together leads to the hypothesis that enhanced left superior parietal activation may reflect increased demand on the sensorimotor control of speech production to avoid errors. This increase is not restricted to the challenges induced by left dorsal striatal damage, but evident in some patient controls with lesions outside the dorsal striatum, in more error prone neurotypical participants, and during performance of more demanding speech production tasks. This suggestion is in line with prior studies that described the parietal somatosensory region as a hub for sensory–motor integration in both macaque and humans (Leh et al. 2007; Passarelli et al. 2021). The parietal somatosensory region is part of a well-established “sensorimotor circuit” that supports motor function by connecting parietal somatosensory and frontal motor cortices to the putamen and then to the lateral thalamus (Alexander et al. 1986). A recent meta-analysis of language studies (Viñas-Guasch and Wu 2017) further characterized this circuit by showing that the left superior parietal lobule and precuneus are functionally connected with the left anterior putamen in healthy human adults performing various language tasks.

Based on our data, we cannot determine if this enhancement of activation is specific to speech production tasks, or to tasks in which the patients, as a group, have impaired performance. Future studies can define the specificity of this enhanced activation by including tasks in which the patient group has intact performance.

### Enhanced left superior parietal activation in post-stroke aphasia

The region of enhanced left parietal activation within the POI group was also activated during speech production in neurotypical adult participants. This adds to previous studies suggesting that aphasia recovery after stroke is frequently supported by undamaged parts of the same system activated by neurotypical adults (Geranmayeh et al. 2014; Stefaniak et al. 2021). Upregulation in the left superior parietal ROI could either be (i) within the striatal–cortical network, driven by residual function in the (partially damaged) striatum, or (ii) in a neural circuit that is independent of the left putamen and compensates when the putamen circuit is dysfunctional. If the former is true, then we would expect that gradual decreases in left dorsal striatum activation would result in proportional increases in left superior parietal activation, in neurotypical participants. Although we found that successful speech production, in error-prone neurotypical participants was associated with higher left superior parietal activation in Experiment 1 and lower left dorsal striatal activation in Experiment 2, we did not observe a significant trade-off in activation between these 2 regions within experiment. Further experiments are therefore required to understand the relationship between left dorsal striatal and left superior parietal activation in both NC and patients with varying degrees of damage to each region, as well as varying degrees of recovery.

Our observation that left superior parietal activation was also enhanced in patients with damage to other left and right hemisphere regions may help to explain why the left superior parietal cortex (along with several other regions) was found, in a meta-analysis, to be hyperactivated during resting state fMRI in patients with aphasia compared with healthy adults (Du et al. 2020). Variability in how the left superior parietal cortex responds to task demands may reflect other factors that influence performance (e.g. age and education) and premorbid differences in functional anatomy. These variables may also explain inconsistent reports of how superior parietal activation during object naming is influenced by speech and language therapy in patients with post-stroke aphasia (Abel et al. 2014; Abel et al. 2015).

### Future directions and study caveats

The main limitation of our study is that, although we studied patients with focal damage to the left dorsal striatum, there was variability in the precise location of the lesions. This variability might explain why we did not find significant group effects in activation and underactivation within the dorsal striatum, despite the fact that each of our POI showed these effects in the individual subject analyses (Fig. 3).

Our results concern patients with left dorsal striatal lesions. Although right striatal damage has not previously been associated with language deficits (cf. Wallesch et al. 1983), a meta-analysis of functional imaging studies in healthy adults suggested that the right putamen is likely to play some role in language processing, even if secondary and more restricted than that of the left (Viñas-Guasch and Wu 2017). Further studies are therefore required to investigate if subtler speech production impairments result from right striatal damage and the recovery patterns that might follow.

Given the size of our POI group we could not examine in this study if damage to distinct parts of the left dorsal striatum (e.g. head of caudate vs. putamen, or anterior vs. posterior putamen) result in differential effects. Precise lesion location might be especially crucial when small complex structures such as the basal ganglia are affected. For example, it is known that the source of input to the putamen (bilaterally) changes when moving along the rostral–caudal axis (reviewed in Ell et al. 2011), and that the functional connectivity pattern for the anterior and posterior putamen differs significantly during language tasks (Viñas-Guasch and Wu 2017). It has also been shown that the posterior putamen is involved in well-learnt responses, whereas the anterior putamen is involved in novel response selection (Jueptner and Weiller 1998; Lehéricy et al. 2005; Bapi et al. 2006). Accordingly, activation in the posterior putamen was previously associated with reading or repeating of words more than pseudowords, whereas activation in the anterior putamen was associated with reading of pseudowords (Oberhuber et al. 2013), and of low-frequency words (Rao and Singh 2015). These effects were observed bilaterally, but were more significant in the left hemisphere. Hence, future studies with larger cohorts are needed to define how damage to different parts of the dorsal striatum affects language recovery and the neural mechanisms underpinning it.

We described patients’ speech impairments early after stroke based on medical notes and retrospective ratings by the patients themselves. Future studies could determine the relationship between speech impairments early after stroke, and functional brain reorganization at the chronic stage, by acquiring standardized behavioral data at the acute stage and following the patients longitudinally.

Our results are also restricted to those patients who were able to participate in this relatively demanding fMRI study. Future studies, using shorter and simpler experimental tasks (e.g. pre-defined single word responses) can examine if the results obtained here are applicable to patients with more severe aphasic symptoms.

Future studies are also needed to establish whether the enhanced left parietal activation documented here is beneficial or detrimental to behavioral performance, by applying noninvasive neurostimulation to the area of enhanced activation in recovered patients. In this sense, significantly higher left parietal activation for correct compared with incorrect spoken responses during sentence production, as we have shown here, does seem to suggest that this region may help to compensate for loss of function following damage to the left dorsal striatum and/or other speech production regions. Finally, the direction of functional connectivity between the left striatum and left superior parietal cortex in both neurotypical and patient populations could be assessed using Dynamic Causal Modeling. These sorts of studies may also allow us to infer whether upregulation in the left superior parietal ROI is driven by (i) residual function in the left striatum; or (ii) a neural circuit that is independent of the left striatum.

### Summary and conclusions

We reported on task-dependent abnormal brain activation in a group of patients with focal lesions to the left dorsal striatum. We found higher than normal activation in the left superior parietal lobe in 2 experiments employing different speech production tasks. By examining the variability within the patient group, across patient groups, across different tasks, and in neurotypical participants, we provide evidence that left superior parietal activation is associated with enhanced speech production demands, with sensitivity to the number of words articulated as well as the nature of the task (verbal > nonverbal). Future longitudinal studies can examine how left superior parietal activation in stroke survivors unfolds over time, whether activation is related to recovery and therapy gains, and importantly, the causal effect of this activation. Altogether, our study enhances our understanding of the neural mechanisms that support language processing in challenging conditions, with stroke affecting language processing regions being one such case.

## Supplementary Material

Geva_SupplementaryMaterial_bhac282
