# Supplementary material for: Enhanced left superior parietal activation during successful speech production in patients with left dorsal striatal damage and error-prone neurotypical participants
Source: Cereb Cortex. 2022 Aug 13;33(7):3437–53. doi: 10.1093/cercor/bhac282 (PMC10068299; doi:10.1093/cercor/bhac282)
Supplement: Geva_SupplementaryMaterial_bhac282 [file geva_supplementarymaterial_bhac282.docx]

# **Supplementary Material**

# Performance on the Comprehensive Aphasia Test

**Table 1. T-scores for the Comprehensive Aphasia Test for patients of interest.**

| **ID** | **Stroke to CAT** | **CAT to fMRI** | **Verbal Fluency** | **Repetition** | | | **Speaking** | | | **Reading** | | | | **Writing** | | **Comp.** | |
| --- | --- | --- | --- | --- | --- | --- | --- | --- | --- | --- | --- | --- | --- | --- | --- | --- | --- |
|  |  |  |  | **Words** | **Complex words** | **Non words** | **Object Naming** | **Action Naming** | **Picture desc.** | **Words** | **Complex words** | **Function words** | **Non words** | **Object naming** | **Picture desc.*** | **Spoken (total)** | **Written (total)** |
| **Cut-off** |  |  | 57 | 56 | 55 | 51 | 61 | 59 | 60 | 61 | 57 | 48 | 56 | 54 | 65 | 56 | 53 |
| **Max** |  |  | 75 | 65 | 62 | 67 | 74 | 69 | 75 | 69 | 67 | 62 | 68 | 67 | 75 | 65 | 62 |
| **PS0619** | 4:6 | 5:5 | **57** | 65 | 62 | 58 | 64 | **59** | 64 | **57** | **40** | 62 | 68 | 55 | **61** | 60 | 65 |
|  | 10:6 | -0:6 | 59 | 65 | 62 | 67 | 64 | 69 | 64 | **60** | **57** | 62 | 68 | 55 | **60** | 58 | **58** |
| **PS0883** | 1:8 | 2:0 | 68 | 65 | 62 | 67 | 74 | 69 | 68 | 62 | 67 | 62 | 68 | 67 | **65** | 63 | 66 |
| **PS2057** | 21:2 | 1:8 | 71 | 65 | 62 | **51** | 74 | 63 | 68 | 69 | 67 | 62 | 68 | 67 | **-** | 62 | 63 |
| **PS2371** | 2:9 | 1:9 | 60 | 65 | 62 | 58 | 70 | 63 | 65 | 69 | 61 | 62 | 68 | 67 | 73 | 60 | 68 |
| **PS2439** | 2:10 | 1:8 | 74 | 65 | 62 | 58 | 66 | 69 | 74 | 69 | 67 | 62 | 68 | 67 | **62** | 63 | 73 |

‘Stroke to CAT’ and ‘CAT to fMRI’ are indicated in [years:months]. PS0619 was tested twice on the CAT, with the second testing occurring after the fMRI scan (indicated as [–] CAT to scan). Impaired scores are in bold and highlighted in colour.

Cut-off score = the highest score within the impaired range; Max score = the highest score that can be obtained on the sub-test; ID = patient ID in the PLORAS database; CAT = Comprehensive Aphasia Test; Picture desc = picture description; Comp = comprehension.

* Written picture description: PS0619: low score is due to low number of appropriate words, high number of inappropriate words, and low score for grammatical well-formedness; PS0883: dominant arm weakness makes writing difficult and slow, resulting in low amount of words; PS2057: patient declined written picture description due to dominant hand weakness and fatigue; PS2439: low score due to limited written output. The patient is able to write with her right hand but this is slower due to mobility problems on her right side.

# Behavioural performance inside the scanner

**Table 2. Correlations between demographic and behavioural variables.**

| ***All participants***  ***(n=125)*** | | **Education** | **Age** | **Lesion volume** | **Time since stroke** | **Accuracy Exp. 1** | **Accuracy Exp. 2** |
| --- | --- | --- | --- | --- | --- | --- | --- |
| **Accuracy Exp. 1** | Correlation | 0.304^*^ | -0.503^*^ |  |  |  |  |
|  | p-value | 0.001 | < 0.001 |  |  |  |  |
| **Accuracy Exp. 2** | Correlation | 0.293^*^ | -0.585^*^ |  |  |  |  |
|  | p-value | 0.001 | < 0.001 |  |  |  |  |
| **RT Exp. 1 (ms)** | Correlation | -0.365^*^ | 0.489^*^ |  |  | -0.746^*^ |  |
|  | p-value | < 0.001 | < 0.001 |  |  | < 0.001 |  |
| **RT Exp. 2 (ms)** | Correlation | -0.352^*^ | 0.339^*^ |  |  |  | -0.673^*^ |
|  | p-value | < 0.001 | < 0.001 |  |  |  | < 0.001 |
| ***NC only***  ***(n=54)*** | | **Education** | **Age** | **Lesion volume** | **Time since stroke** | **Accuracy Exp. 1** | **Accuracy Exp. 2** |
| **Accuracy Exp. 1** | Correlation | 0.185 | -0.434* |  |  |  |  |
|  | p-value | 0.180 | 0.001 |  |  |  |  |
| **Accuracy Exp. 2** | Correlation | 0.268 | -0.442* |  |  |  |  |
|  | p-value | 0.050 | 0.001 |  |  |  |  |
| **RT Exp. 1 (ms)** | Correlation | -0.259 | 0.400* |  |  | -0.354* |  |
|  | p-value | 0.059 | 0.003 |  |  | 0.009 |  |
| **RT Exp. 2 (ms)** | Correlation | 0.033 | 0.128 |  |  |  | -0.274* |
|  | p-value | 0.814 | 0.357 |  |  |  | 0.045 |
| ***Patients only***  ***(n=71)*** | | **Education** | **Age** | **Lesion volume** | **Time since stroke** | **Accuracy Exp. 1** | **Accuracy Exp. 2** |
| **Accuracy Exp. 1** | Correlation | 0.005 | -0.143 | -0.363^*^ | -0.190 |  |  |
|  | p-value | 0.965 | 0.235 | 0.002 | 0.112 |  |  |
| **Accuracy Exp. 2** | Correlation | -0.062 | -0.344^*^ | -0.295 | -0.076 |  |  |
|  | p-value | 0.608 | 0.003 | 0.013 | 0.528 |  |  |
| **RT Exp. 1 (ms)** | Correlation | -0.092 | 0.171 | 0.389^*^ | 0.099 | -0.606^*^ |  |
|  | p-value | 0.446 | 0.153 | 0.001 | 0.410 | < 0.001 |  |
| **RT Exp. 2 (ms)** | Correlation | -0.250* | 0.114 | 0.252 | 0.043 |  | -0.450^*^ |
|  | p-value | 0.036 | 0.342 | 0.034 | 0.723 |  | < 0.001 |

Accuracy = percent of correct trials. Correlations are reported using Spearman’s rho. 2-tailed p-values are reported for all. Highlighted values (coloured and marked by asterisk) are significant after FDR correction for multiple comparisons.

## Experiment 1

A 3x3 MANOVA of **speech production** **accuracy level** revealed a significant main effect of group (F_(122,2)_ = 35.58, p < 0.001), task (F_(122,2)_ = 33.03, p < 0.001), and a significant interaction between task and group (F_(122,4)_ = 16.92, p < 0.001). Similarly, a 3x2 MANOVA of **semantic decision** **accuracy level** revealed a significant main effect of group (F_(122,2)_ = 6.64, p = 0.002), no effect of task (F_(122,1)_ = 2.11, p = 0.149), but a trend towards interaction between task and group (F_(122,2)_ = 2.94, p = 0.057). Post-hoc tests showed that there was no significant difference in accuracy between patients of interest and neurotypical controls or patient controls (p > 0.05 for all). In fact, the significant effects were driven by the lower accuracy of the patient controls relative to the neurotypical controls in all five tasks (p < 0.05 for all).

A 3x3 MANOVA of **speech production** **response times** (performed only for the three speech production tasks), revealed a significant main effect of group (F_(120,2)_ = 52.69, p < 0.001), task (F_(122,2)_ = 24.17, p < 0.001), and a significant interaction between task and group (F_(122,4)_ = 8.38, p < 0.001). Post-hoc tests showed that both patient groups (POI and PC) were slower than the NC group on all three tasks (P < 0.05 for all), with the two patient groups differing only on the sentence production task (PC were significantly slower than POI, t_(13.89)_ = 5.09, p < 0.001).

In summary, in Experiment 1, POI had overall intermediate performance (with NC having higher accuracy scores and PC lower accuracy scores), but these differences were not statistically significant. However, both patient groups were significantly slower than NC on all five tasks (see Supplementary Figure 1 and Table 3 below).

**Figure 1. Mean accuracy and response time in Experiment 1.**

**
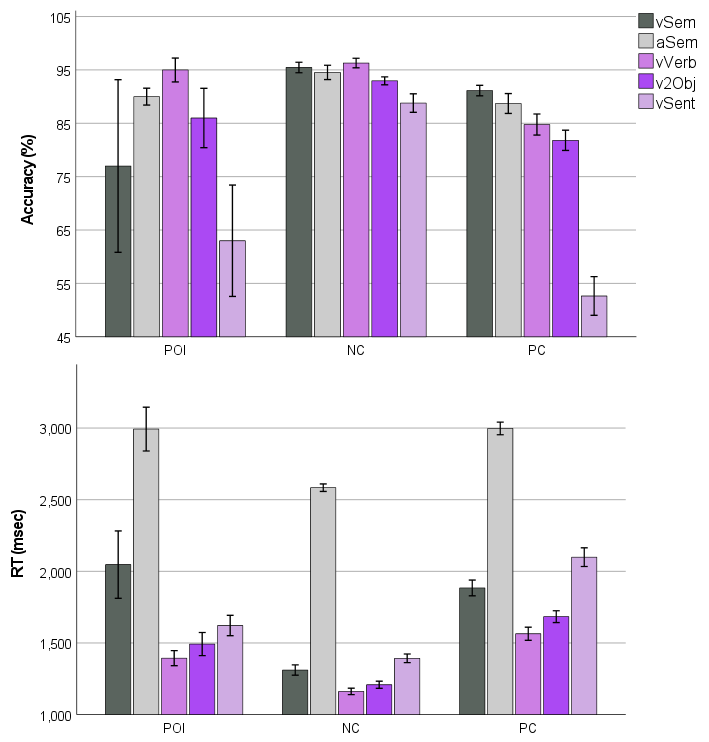
**

Accuracy (percent correct; top) and response times (RT in ms; bottom) during different tasks (semantic decision = grey / speech production = purple). Error bars represent ±1 standard error. fMRI Tasks, from left to right: visual semantic decision (vSem); auditory semantic decision (aSem); naming two visual objects (v2Obj); verb production from visual picture (vVerb); sentence production from visual picture (vSent). NC = neurotypical controls, PC = all patient controls, POI = patients of interest, RT = response time.

**Table 3. Individual and group performance on the fMRI tasks in Experiment 1.**

**a.** Accuracy (percent correct)

| **ID / Group** | **vSem** | **aSem** | **vVerb** | **v2Obj** | **vSent** |
| --- | --- | --- | --- | --- | --- |
| **POI** |  |  |  |  |  |
| PS0619 | 15 | 90 | 90 | 65 | 35 |
| PS0883 | 100 | 90 | 100 | 95 | 80 |
| PS2057 | 75 | 95 | 90 | 95 | 40 |
| PS2371 | 95 | 85 | 95 | 90 | 80 |
| PS2439 | 100 | 90 | 100 | 85 | 80 |
| Mean | 77.0 | 90.0 | 95.0 | 86.0 | 63.0 |
| SD | 36.2 | 3.5 | 5.0 | 12.4 | 23.3 |
| **PC** |  |  |  |  |  |
| Mean | 91.1 | 88.7 | 84.8 | 81.8 | 52.7 |
| SD | 8.0 | 15.1 | 16.0 | 15.4 | 29.4 |
| Min | 65.0 | 15.0 | 15.0 | 25.0 | 5.0 |
| Max | 100.0 | 100.0 | 100.0 | 100.0 | 100.0 |
| **NC** |  |  |  |  |  |
| Mean | 95.5 | 94.5 | 96.3 | 93.0 | 88.8 |
| SD | 7.2 | 9.9 | 6.6 | 5.5 | 12.7 |
| Min | 55.0 | 30.0 | 70.0 | 80.0 | 35.0 |
| Max | 100.0 | 100.0 | 100.0 | 100.0 | 100.0 |

**b.** Average response time across trials (ms)

| **ID / Group** | **vSem** | **aSem** | **vVerb** | **v2Obj** | **vSent** |
| --- | --- | --- | --- | --- | --- |
| **POI** |  |  |  |  |  |
| PS0619 | 2891 | 3204 | 1507 | 1581 | 1707 |
| PS0883 | 1527 | 2421 | 1414 | 1259 | 1380 |
| PS2057 | 1761 | 3107 | 1485 | 1743 | 1770 |
| PS2371 | 1886 | 3279 | 1216 | 1434 | 1547 |
| PS2439 | 2172 | 2954 | 1349 | 1447 | 1708 |
| Mean | 2047 | 2993 | 1394 | 1493 | 1622 |
| SD | 526 | 342 | 117 | 181 | 159 |
| **PC** |  |  |  |  |  |
| Mean | 1881 | 2995 | 1565 | 1689 | 2121 |
| SD | 440 | 353 | 368 | 331 | 550 |
| Min | 1200 | 2347 | 939 | 1158 | 1153 |
| Max | 3647 | 4530 | 2881 | 3204 | 3521 |
| **NC** |  |  |  |  |  |
| Mean | 1312 | 2589 | 1164 | 1217 | 1394 |
| SD | 258 | 189 | 161 | 189 | 214 |
| Min | 880 | 2250 | 855 | 759 | 810 |
| Max | 2212 | 3291 | 1639 | 2013 | 1972 |

Highlighted are accuracy scores lower than the minimum score in the NC group; and response times higher than maximum RT in the NC group. NC = neurotypical controls, PC = all patient controls, POI = patients of interest, SD = standard deviation. See Supplementary Figure 1 above for task abbreviations.

## Experiment 2

A 3x2x2x2 MANOVA of **accuracy** **level** revealed a significant main effect of group (F_(122,2)_ = 33.42, p < 0.001), semantic input (F_(122,1)_ = 4.43, p = 0.037), verbal input (F_(122,1)_ = 20.60, p < 0.001), and a trend towards interaction between verbal input and group (F_(122,2)_ = 2.69, p = 0.072), but no other significant main effects or interactions (p > 0.05 for all). Post-hoc tests showed that there was no significant difference in overall accuracy, or accuracy in the verbal conditions, between patients of interest and neurotypical controls (p > 0.05 for both). The significant effects were driven by the lower accuracy of the patient controls relative to the neurotypical controls in all conditions combined (t_(73.95)_ = 8.88, p < 0.001), and relative to the patients of interest in the verbal condition (t_(9.63)_ = 3.43, p = 0.007).

A 3x2x2x2 MANOVA of **response times** measured during correct trials showed a significant main effect of group (F_(116,2)_ = 38.94, p < 0.001), and of verbal input (F_(116,1)_ = 172.83, p < 0.001). All other main and interaction effects were not significant (p > 0.05 for all). Similar to the accuracy scores, post-hoc tests showed that patients of interest were not slower than neurotypical controls in either overall RT, or average RT across the verbal conditions (p > 0.05 for both). Again, the main group effect was driven by the slower response of the patient controls relative to the neurotypical controls (average RT across all tasks, p < 0.001 for all comparisons), and relative to the patients of interest (average RT across the verbal tasks, t_(69)_ = 2.19, p = 0.032).

In summary, in Experiment 2, patients of interest did not differ in performance from neurotypical controls, while patient controls were slower and less accurate than both POI and NC on various conditions (see Supplementary Figure 2 and Table 4 below).

**Figure 2. Mean accuracy and response time in Experiment 2.**


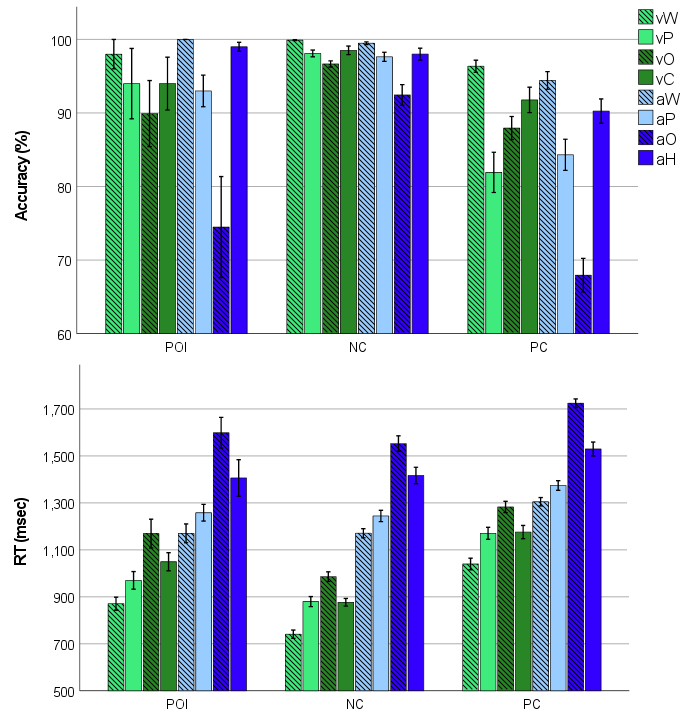


Accuracy (percent correct; top) and response times (RT in ms; bottom) during tasks differing in stimulus modality (visual = green / auditory = blue), verbal input (verbal = light colour / non-verbal = dark colour), and semantic input (semantic = stripes / non-semantic = no stripes). Error bars represent ±1 standard error. fMRI Tasks, from left to right: word reading (vW); pseudoword reading (vP); object naming (vO); colour naming (vC); word repetition (aW); pseudoword repetition (aP); auditory object naming (aO); naming gender of humming voice (aH). NC = neurotypical controls, PC = all patient controls, POI = patients of interest, RT = response time.

**Table 4. Individual and group performance on the fMRI tasks in Experiment 2.**

**a.** Accuracy (percent correct)

| **ID / Group** | **vW** | **vP** | **vO** | **vC** | **aW** | **aP** | **aO** | **aH** |
| --- | --- | --- | --- | --- | --- | --- | --- | --- |
| **POI** |  |  |  |  |  |  |  |  |
| PS0619 | 90 | 75 | 75 | 80 | 100 | 95 | 55 | 98 |
| PS0883 | 100 | 98 | 100 | 100 | 100 | 100 | 88 | 100 |
| PS2057 | 100 | 100 | 85 | 95 | 100 | 88 | 90 | 98 |
| PS2371 | 100 | 100 | 95 | 98 | 100 | 93 | 63 | 100 |
| PS2439 | 100 | 98 | 95 | 98 | 100 | 90 | 78 | 100 |
| Mean | 98.0 | 94.0 | 89.9 | 94.0 | 100.0 | 93.0 | 74.5 | 99.0 |
| SD | 4.5 | 10.7 | 10.0 | 8.0 | 0.0 | 4.8 | 15.4 | 1.4 |
| **PC** |  |  |  |  |  |  |  |  |
| Mean | 96.4 | 81.9 | 88.0 | 91.8 | 94.4 | 84.3 | 68.0 | 90.3 |
| SD | 6.7 | 22.2 | 12.8 | 14.0 | 9.7 | 17.2 | 18.6 | 13.4 |
| Min | 62.5 | 20.0 | 30.0 | 27.5 | 45.0 | 10.0 | 20.0 | 27.5 |
| Max | 100.0 | 100.0 | 100.0 | 100.0 | 100.0 | 100.0 | 97.5 | 100.0 |
| **NC** |  |  |  |  |  |  |  |  |
| Mean | 99.9 | 98.1 | 96.7 | 98.5 | 99.5 | 97.6 | 92.5 | 98.0 |
| SD | 0.5 | 3.4 | 3.1 | 4.2 | 1.3 | 4.6 | 10.3 | 5.9 |
| Min | 98 | 85 | 88 | 75 | 95 | 83 | 55 | 60 |
| Max | 100 | 100 | 100 | 100 | 100 | 100 | 100 | 100 |

**b.** Average response time across trials (ms)

| **ID / Group** | **vW** | **vP** | **vO** | **vC** | **aW** | **aP** | **aO** | **aH** |
| --- | --- | --- | --- | --- | --- | --- | --- | --- |
| **POI** |  |  |  |  |  |  |  |  |
| PS0619 | 961 | 1072 | 1245 | 1088 | 1115 | 1207 | 1613 | 1451 |
| PS0883 | 830 | 904 | 1073 | 970 | 1104 | 1155 | 1685 | 1543 |
| PS2057 | 900 | 1051 | 1359 | 1155 | 1310 | 1333 | 1496 | 1242 |
| PS2371 | 861 | 902 | 1016 | 951 | 1111 | 1257 | 1417 | 1207 |
| PS2439 | 803 | 922 | 1155 | 1084 | 1212 | 1339 | 1784 | 1590 |
| Mean | 871 | 970 | 1170 | 1050 | 1171 | 1258 | 1599 | 1406 |
| SD | 62 | 84 | 137 | 86 | 90 | 80 | 146 | 174 |
| **PC** |  |  |  |  |  |  |  |  |
| Mean | 1042 | 1175 | 1283 | 1178 | 1311 | 1387 | 1721 | 1524 |
| SD | 197 | 201 | 187 | 222 | 142 | 178 | 149 | 235 |
| Min | 659 | 755 | 971 | 784 | 1039 | 1094 | 1382 | 1018 |
| Max | 1729 | 1711 | 1688 | 1797 | 1703 | 1932 | 2029 | 2165 |
| **NC** |  |  |  |  |  |  |  |  |
| Mean | 745 | 883 | 991 | 886 | 1171 | 1252 | 1547 | 1408 |
| SD | 128 | 157 | 149 | 137 | 138 | 178 | 236 | 259 |
| Min | 515 | 535 | 575 | 604 | 827 | 825 | 837 | 813 |
| Max | 1068 | 1274 | 1272 | 1405 | 1519 | 1635 | 2086 | 1941 |

Highlighted are accuracy scores lower than the minimum score in the NC group; and response times higher than maximum RT in the NC group. NC = neurotypical controls, PC = all patient controls, POI = patients of interest, SD = standard deviation. See Supplementary Figure 2 above for task abbreviations.

# Dorsal-striatal speech production activation

**Figure 3. Dorsal-striatal activation in patients of interest in Experiment 2.**


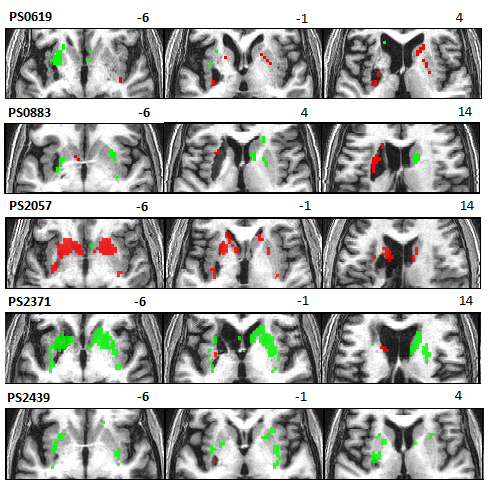


Thresholded speech production activation in Experiments 2, displayed within the boundaries of the dorsal striatum bilaterally. Activation (Green) is derived from each participant’s 1^st^ level analysis, for the contrast [8 Tasks – Rest]. Under-activation (Red) is derived from the 2^nd^ level analysis, for the contrast [NC group > each patient]. Activations are presented at a voxel-level statistical threshold of p < 0.05 FWE-corrected across the whole brain, other than [NC > PS2439] in Experiment 2, which is displayed at a voxel-level statistical threshold of p < 0.001 uncorrected. Patient’s ID numbers from the PLORAS database and z coordinates are displayed above the images.

# Activation outliers among patients of interest

We identified two outliers among POI which might have driven the effects of interest: in Experiment 1, PS0619 had extremely high activation in the sentence production task (vSent), and in Experiment 2, the same participant had extremely low activation in the object naming task (vO). See datapoints highlighted in red in Figure 6. To examine whether effects in the parietal ROI were driven solely by this outlier, we excluded the outlier from the analysis, and the results remained similar, see Table 5 below.

**Table 5. Left superior parietal activation among four patients of interest (excluding PS0619).**

| **Region** | **Condition** | **Peak coordinates** | | | **Cluster size** | **Z-score** | | |
| --- | --- | --- | --- | --- | --- | --- | --- | --- |
|  |  |  |  |  |  | **POI >**  **NC** | **POI >**  **PC-nostr** | **POI >**  **PC-str** |
|  |  | **x** | **y** | **z** |  |  |  |  |
| ROI-PAR_1_  (Exp. 1) | 3 speech production tasks - Rest | -3 | -37 | 62 | 139 | 4.79 | 3.59* | 3.66* |
|  |  | -21 | -40 | 65 |  | 4.23* | ns | ns |
|  |  | -9 | -43 | 74 |  | 4.06* | ns | ns |
|  | 3 speech production > 2 semantic tasks | -3 | -37 | 62 | 174 | 5.01 | 3.75* | 3.55* |
|  |  | -21 | -40 | 62 |  | 4.97 | 3.65* | 3.37* |
|  |  | -9 | -43 | 74 |  | 4.15* | 3.15* | ns |
| ROI-PAR_1_  (Exp. 2) | 8 speech production tasks - Rest | -12 | -43 | 74 | 12 | 4.41* | 3.31* | 3.30* |
|  | Verbal input - Rest | -12 | -43 | 74 | 15 | 5.28 | 4.11* | 4.31* |
|  | Verbal > Non-verbal input |  |  |  | 3 | 4.04* | 3.35* | 3.88* |

Anatomical and statistical details of the left superior parietal ROI where activation for speech production compared to semantic decision was significantly greater for four POI than NC in Experiment 1 (ROI- PAR_1_, Exp. 1). Z-scores are significant at a voxel-level threshold of p < 0.05 FWE-corrected for multiple comparisons across the whole brain, unless marked with an asterisk (p < 0.001 uncorrected). Peak coordinates are in MNI space; cluster size in number of voxels is reported with a voxel-level statistical threshold of p < 0.001 uncorrected.

# Normal function of the ROIs

**Figure 4. Activation during successful speech production in neurotypical controls.**


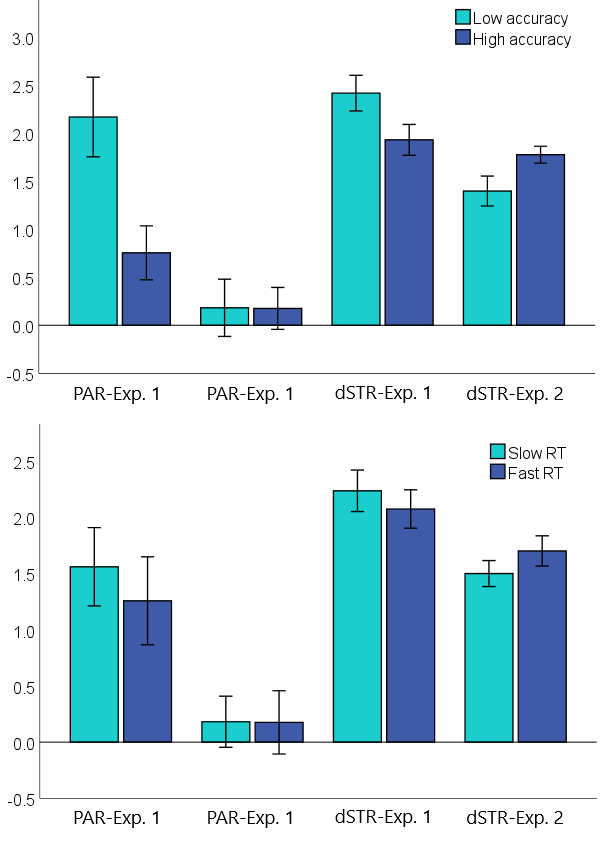


Activation in the parietal region (PAR; see Table 3 for peak coordinates) and dorsal striatum (dSTR, see Table 4 for peak coordinates), among neurotypical controls with High versus Low Accuracy (Top) or Fast versus Slow RT (Bottom). Y axis represents activation (mean of the principal eigenvariate within a 3 mm radius sphere centred on the peak coordinate). Eigenvariates and accuracy were averaged across the three speech production tasks in Experiment 1, or the four speech production tasks with verbal input in Experiment 2.

# Activation associated with correct versus incorrect trials

To compare activation for correct and incorrect trials, we performed a 3x2 ANOVA (3 Groups: NC, PC, POI; 2 response types: correct and incorrect) using data from the sentence production task (vSent) which was the only speech production task that had more than 1 incorrect trial in all 5 POI (see accuracy scores in Supplementary Tables 3 and 4 above).

Activation for correct > incorrect trials was significant across all participants who made sentence production errors, and in each of the groups (NC, PC, POI), in both ROIs (ROI-PAR_1_ Exp. 1 and ROI-PAR_2_ Exp. 2), except for PC in ROI-PAR_2_ Exp. 2 (statistical height threshold of p < 0.05 FWE-corrected within each ROI). See Supplementary Table 6 below. There was no significant activation for incorrect > correct trials, in any of the groups or ROIs (p > 0.05 uncorrected).

We note that higher activation for correct than incorrect trials could be due to inefficient estimation of activation related to incorrect trials, due to the overall smaller number of incorrect trials. To account for this, we compared activation for correct and incorrect trials (i) in a subgroup of participants who had ≥5 incorrect/correct trials (out of the 20 trials; n = 33; see Supplementary Table 6 below); and, (ii) using within subject design (paired sample t-test), in a subgroup of participants who had around 50% accuracy (8-12 correct / incorrect trials out of 20 trials; n = 17, see Supplementary Table 6 below). Results in both analyses support our findings above, as in all cases significant activation was found for correct > incorrect trials, but not vice versa.

**Table 6. Activation associated with correct > incorrect trials (sentence production, Exp. 1)**

| **ROI** | **Group** | **n** | **Peak coordinates** | | | **Cluster size** | **Z-score** | **p-value** |
| --- | --- | --- | --- | --- | --- | --- | --- | --- |
|  |  |  | **x** | **y** | **z** |  |  |  |
| > 1 incorrect / correct response | |  |  |  |  |  |  |  |
| ROI-PAR_1_ (Exp. 1) | Main effect | 110 | -18 | -37 | 62 | 58 | 3.74 | 0.003 |
|  | NC | 42 | -18 | -37 | 68 | 40 | 2.89 | 0.041 |
|  | PC | 63 | -18 | -40 | 56 | 17 | 2.91 | 0.040 |
|  | POI | 5 | -18 | -37 | 59 | 42 | 2.99 | 0.032 |
| ROI-PAR_2_ (Exp. 2) | Main effect | 110 | -18 | -37 | 68 | 20 | 3.40 | 0.005 |
|  | NC | 42 | -18 | -37 | 68 | 9 | 2.89 | 0.021 |
|  | PC | 63 | -15 | -43 | 71 | 4 | 2.13 | 0.133* |
|  | POI | 5 | -15 | -40 | 68 | 17 | 2.72 | 0.034 |
| ≥ 5 incorrect / correct response | |  |  |  |  |  |  |  |
| ROI-PAR_1_ (Exp. 1) | Patients | 33  (31 PC, 2 POI) | -12 | -40 | 62 | 52 | 3.80 | 0.003 |
| ROI-PAR_2_ (Exp. 2) | Patients | 33  (31 PC, 2 POI) | -15 | -40 | 71 | 18 | 3.14 | 0.011 |
| ~50% (8 – 12) correct / incorrect responses | | | |  |  |  |  |  |
| ROI-PAR_1_ (Exp. 1) | PC | 17  (16 PC, 1POI) | -27 | -40 | 68 | 49 | 3.93 | 0.003 |
| ROI-PAR_2_ (Exp. 2) | PC | 17  (16 PC, 1POI) | -15 | -37 | 65 | 15 | 2.77 | 0.038 |

Peak coordinates are in MNI space; p-value FWE-corrected within the ROI; Asterisk denotes non-significant result.
